# Postoperative pain intensity after using different instrumentation techniques: a randomized clinical study

**DOI:** 10.1590/1678-77572016-0138

**Published:** 2017

**Authors:** Ersan ÇIÇEK, Mustafa Murat KOÇAK, Sibel KOÇAK, Baran Can SAĞLAM, Sevinç Aktemur TÜRKER

**Affiliations:** 1 Bülent Ecevit University, Faculty of Dentistry, Department of Endodontics, Zonguldak, Turkey.

**Keywords:** Endodontics, Instrumentation, Postoperative pain

## Abstract

Postoperative pain is a frequent complication associated with root canal treatment, especially during apical instrumentation of tooth with preexisting periradicular inflammation

**Objectives:**

The aim of this clinical study was to evaluate the influence of the instrumentation techniques on the incidence and intensity of postoperative pain in single-visit root canal treatment.

**Material and Methods:**

Ninety patients with single root/canal and non-vital pulps were included. The patients were assigned into 3 groups according to root canal instrumentation technique used; modified step-back, reciprocal, and rotational techniques. Root canal treatment was carried out in a single visit and the severity of postoperative pain was assessed via 4-point pain intensity scale. All the participants were called through the phone at 12, 24 and 48 h to obtain the pain scores. Data were analyzed through the Kruskal–Wallis test.

**Results:**

There was significant difference between all groups (p<0.05). The modified step-back technique produced postoperative pain significantly lower than the rotational (p=0.018) and reciprocal (p=0.020) techniques. No difference was found between the reciprocal and rotational techniques (p=0.868). Postoperative pain in the first 12 h period (p=0.763) and in the 24 h period (p=0.147) was not significantly different between the groups. However, the difference in the 48 h period was statistically different between the groups (p=0.040).

**Conclusion:**

All instrumentation techniques caused postoperative pain. The modified step-back technique produced less pain compared to the rotational and reciprocal techniques.

## Introduction

Postoperative pain is a frequent complication associated with root canal treatment, and can be influenced by insufficient root canal preparation, extrusion of irrigant, debris or intracanal interappointment medicament, presence of preoperative pain, presence of periapical pathosis, and apical patency during root canal instrumentation^1,22^. The apical extrusion of irrigant and debris, including bacteria and necrotic tissue, may lead to postoperative pain, periapical inflammation and postoperative flare-ups^23^. Even though all instrumentation techniques and instruments are associated with debris extrusion, the instrumentation techniques and the design of the files may affect the amount of debris extrusion^6^.

Recent studies have demonstrated that reciprocating systems can produce extrusion of debris in the apical region, which could be related with postoperative pain when compared with other traditional instrumentation techniques^8,15^. Reciprocating motion may increase the amount of debris extruded beyond the apex and consequently the risk of postoperative pain compared to rotary instrumentation^5,6^. In a recent randomized, double-blind study, Shokraneh, et al.^24^ (2016) demonstrated that postoperative pain was significantly lower in patients undergoing root canal instrumentation with the Wave-One file compared with the ProTaper Universal and hand files.

ProTaper Next (Dentsply Maillefer, Ballaigues, Switzerland) is manufactured from M-Wire nickel-titanium (Ni-Ti) alloy to enhance flexibility and cyclic fatigue resistance. This system is designed with progressive and regressive percentage tapers, and an off-centered rectangular cross section for superior strength to improve canal shaping efficiency^4,12^.

The recently introduced instrumentation system WaveOne (Dentsply Maillefer, Ballaigues, Switzerland) is claimed to be able to completely prepare root canals with a single instrument. The WaveOne instruments work in a reciprocal motion. They are manufactured from M-wire Ni-Ti alloy. This system has potential advantages such as reduced number of instruments and the elimination of cross-contamination depending on the single use of these instruments^15,26^.

In general, postoperative pain begins within a few hours after root canal procedures and frequently requires unscheduled visits^22^. Although the reasons for such exacerbations are not well-known, changes in periapical pressure, in the number or virulence of endodontic microbiota, or in environmental conditions may be possible reasons^25^.

The root canal treatment of tooth with necrotic pulp and apical periodontitis can be completed in single or multiple visits. Clinical studies demonstrated that patients generally tolerate and prefer single-visit root canal treatment^21^ because of several advantages, such as reduction of operative procedures^17^, no inter-appointment leakage^3^, being less time consuming and more economical^28^.

The aim of the present study was to compare the incidence and intensity of postoperative pain related to different root canal instrumentation techniques during single visit root canal treatment. The null hypothesis of this study was that the instrumentation technique does not affect the intensity of postoperative pain.

## Material and methods

This clinical study was performed under the regulations of the ethics committee (protocol number: 2013-116-01/10). The project was registered at www.clinicaltrial.gov (ClinicalTrials.gov Identifier: NCT02566486). Asymptomatic necrotic maxillary and mandibular teeth which had single straight root canal with apical radiolucency and periodontal probing of at most 3 mm depth were included in the study. On the other hand, patients with sinus tract, periapical abscess or facial cellulitis, who had previously taken any medication, and who had tooth with any type of previous root canal treatment were excluded.

The patients had no symptoms before treatment and were in good health, as determined from a written health history and oral interview. Age, gender, tooth location, pulp sensibility, and radiographically visible lesions (teeth with loss of lamina dura and apically periodontal ligament enlargement of >2 mm were classified as having lesions of endodontic origin^19^) were recorded. Thermal test and an electric pulp-testing device (Elements pulp vitality tester, SybronEndo, Orange, CA, USA) were used to assess the sensibility of the pulp.

Overall, 90 patients who had asymptomatic, non-vital teeth associated with periapical lesions were included. None of the teeth were tender to percussion and palpation. The ages of patients ranged between 21 and 65 years. An informed written consent in full accordance with the ethical principles was obtained from each patient before root canal treatment was initiated. All diagnoses and treatment procedures were performed by a single operator to eliminate or minimize individual variability in the treatment between clinicians. The patients were randomly allocated to 3 groups of 30 through coin toss. Allocation was performed by a second operator blinded to the treatment procedure. The groups were as follows;

Modified step-back technique (n=30): The canals were instrumented with a modified step-back technique using stainless-steel hand files (Dentsply Maillefer, Ballaigues, Switzerland). A minimal preliminary instrumentation was performed using a 15/.02 hand file. During the modified step-back preparation, the coronal two-thirds of root canals were flared with size 2 and 3 gates-glidden burs after minimal preliminary instrumentation. The root canals were prepared to a master apical size of 40/.02 with K files. Step back technique was performed by using K-files size 45-55/.02. The depth of insertion of each larger file was reduced by 1 mm. continuously a size 40/.02 file after each step-back file was used to control the patency of the apical portion.

Reciprocal technique (n=30): The canals were instrumented with an engine driven reciprocation motion, using a WaveOne file with a size of 40/.08 in a slow in-and-out pecking motion within a 3 mm amplitude limit combined with a brushing motion. The flutes of the instrument were cleaned after 3 pecks.

Rotational technique (n=30): The canals were instrumented using ProTaper Next 40/.06 file in the sequence X1, X2, X3, and X4 at a rotational speed of 300 rpm and 200 g/cm torque according to the manufacturer’s instructions. The instruments were used up to the working length.

After isolation and access cavity preparation, in all groups apical patency was established and confirmed with a size 10 K-File 0.5 mm beyond the apex. The initial working length was then determined with an electronic root canal measurement device (Root ZX mini; J. Morita, Tokyo, Japan). A size 15 K-file, attached to the file clip of the electronic root canal measurement device, was advanced apically into the canal until the screen showed zero. The length of the file was measured and 0.5 mm was subtracted to get to the initial working length which was confirmed using periapical radiographs. During instrumentation, a total of 10 ml of 5.25% NaOCl were used for irrigation. The irrigation needle (NaviTip 31 gauge needle; Ultradent, South Jordan, UT, USA) was placed as deep as possible into the canal without resistance until it was 1 mm shorter than the predetermined working length. The final irrigation was performed with 5 ml 5.25% NaOCl, 5 ml 17% EDTA, and 5 ml 2% chlorhexidine. To prevent any reaction between NaOCl, EDTA and chlorhexidine, 5 ml distilled water was used following each irrigation solution.

The root canals were obturated with gutta-percha and an epoxy-resin based sealer (AH26, De Trey Dentsply, Konstanz, Germany) using cold lateral compaction technique. A standardized gutta-percha master cone size 40/.02 was fitted at the working length. The gutta-percha cone was lightly coated with the sealer and slowly inserted into the canal. Then, a size 20 finger spreader was inserted, rotated, and withdrawn; a size 15/.02 gutta-percha accessory cone, coated with a thin layer of the sealer, was placed into the space created by the spreader. Cold lateral compaction with accessory gutta-percha cones was performed until the cones could not be inserted more than 5 mm into the root canal. After radiographic confirmation of the obturation, coronal seal was provided with a dental adhesive and composite resin, and occlusion was checked. No occlusal reduction was performed. All canals were shaped, cleaned, and obturated in a single visit. No systemic medication for postoperative pain was prescribed.

The assessment of postoperative pain was carried out at 12, 24, and 48 hours after treatment by one independent clinician blinded to the groups. All participants were called through the phone at 12, 24 and 48 h to obtain the pain scores using a 4-point pain intensity scale^7^. The presence, absence, and degree of pain were recorded. The pain categories were as follows:

1. no pain;
2. slight pain (mild discomfort, no need for treatment);
3. moderate pain (pain relieved by analgesics);
4. severe pain (pain and/or swelling not relieved by simple analgesics and unscheduled visit required).

### Outcome measures

Primary outcome; the assessment of postoperative pain was carried out at 12, 24, and 48 hours after the initial appointment by using a 4-point pain intensity scale after the treatment. Secondary outcome; having an unscheduled appointment for emergency treatment or any complications such as postoperative swelling or paresthesia.

All these measures were recorded in the patient’s chart.

### Statistical analysis

Statistical analysis was performed with the SPSS 19.0 software (SPSS Inc., Chicago, IL, USA). Descriptive statistics were expressed as mean and standard deviation. The normality of the data was analyzed with the Kolmogorov-Smirnov test. Differences among the groups were analyzed through the Kruskal–Wallis test. Repeated measures were evaluated with the Friedman test. *P* value of less than 0.05 was considered statistically significant for all tests.

## Results

**Figure 1:**
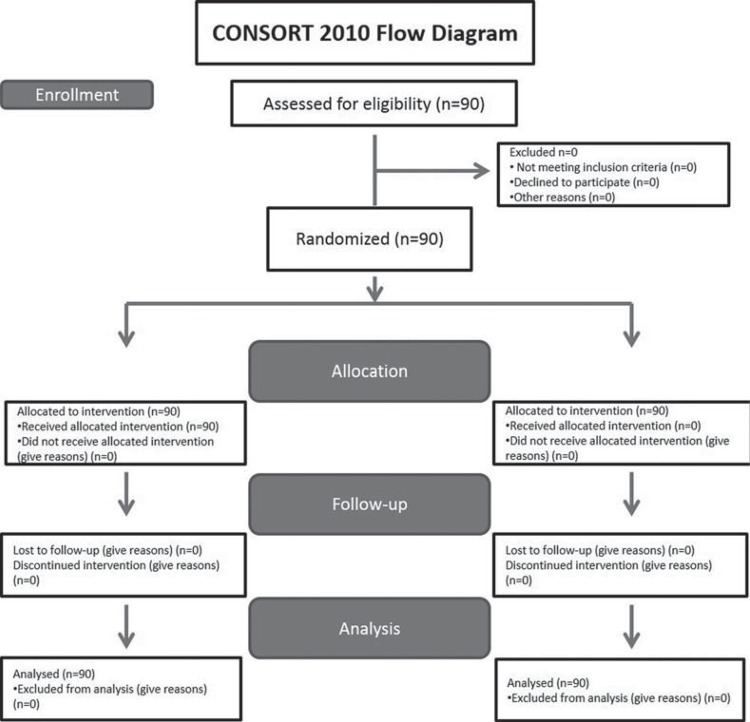
The CONSORT flow diagram of patients enrolled in the clinical trial

The patients enrolled in the clinical trial are presented in the flow diagram (Figure 1). Demographic data are shown in Table 1. All treated teeth were asymptomatic and associated with periapical lesion. None of the patients required an unscheduled appointment for emergency treatment. No patient reported any other symptoms or complications such as postoperative swelling or paresthesia. The intensity of postoperative pain is shown in Table 2. The pain categories ranged between 1 (no pain) and 4 (severe pain). Severe pain was reported only in the first 12 hours. The incidence and intensity of postoperative pain in all groups gradually reduced over the study period. The percentages found in each score assigned to each group are shown in Table 3.

**Table 1:** Baseline demographic characteristic of patients in each group

Baseline demographic characteristic		Modified step-back technique (n=30)	Reciprocal technique (n=30)	Rotational technique (n=30)	P-value
Gender	Male (%)	16 (53.33)	15 (50)	16 (53.33)	0.957*
	Female (%)	14 (46.67)	15 (50)	14 (46.67)	
Age	Mean	35.6	39.6	36	0.10*
	Range	21-56	22-63	21-65	
Location	Maxillary	9 (30)	13 (43.33)	12 (40)	0.42*
	Mandibular	21 (70)	17 (56.66)	18 (60)	

*P>0.05 statistically not significant

**Table 2:** Pain intensity associated with different instrumentation techniques at different time intervals

Group	12 h	24 h	48 h
	Mean ± SD	Mean ± SD	Mean ± SD
Modified step-back technique	1.4 ± 0.563^aA^	1.2 ± 0.407^bB^	1.07 ± 0.254^cC^
Reciprocal technique	1.67 ± 0.959^dA^	1.53 ± 0.730^dB^	1.40 ± 0.675^dD^
Rotational technique	1.57 ± 0.858^eA^	1.43 ± 0.626^eB^	1.33 ± 0.547^eD^

Within the same column, means with different uppercase superscript letter are statistically different (P<0.05)

Within the same row, means with different lowercase superscript letter are statistically different (P<0.05)

**Table 3:** The percentages in each score assigned to each group for different time intervals

Group	12 h				24 h				48 h			
	1	2	3	4	1	2	3	4	1	2	3	4
Modified step-back technique	63.3	33.3	3.34	0	80	20	0	0	93.3	6.67	0	0
Reciprocal technique	60	20	13.3	6.67	60	26.6	13.3	0	70	20	10	0
Rotational technique	60	30	3.33	6.67	63.3	30	6.67	0	70	26.6	3.33	0

The modified step-back technique demonstrated significantly lower postoperative pain than the rotational (p=0.018) and reciprocal techniques (p=0.020). However, no difference was found between the rotational and reciprocal techniques (p=0.868). Postoperative pain was not significantly different in the 12 h (p=0.763) and 24 h periods (p=0.147) between the instrumentation techniques. However, postoperative pain in the 48 h period was statistically different between all groups (p=0.04).

In the modified step-back technique, the differences between the time periods were significant (p=0.001). However, no difference was found between the rotational (p=0.120) and reciprocal techniques (p=0.191) in terms of time periods.

## Discussion

Several factors including age, sex, pulpal and periradicular status, type of tooth, preoperative pain, and technical aspects may affect the occurrence of postoperative pain^29^. Among these factors only technical aspects including instrumentation, irrigation, and obturation protocols are under the operator’s control. Therefore, severe postoperative pain can be avoided by providing a well-cleaned and shaped canal and by minimizing the extrusion of canal contents during the process^11^. Several factors, such as preoperative diagnosis, the ability to obtain infection control, root canal anatomy, procedural complications, and subjective factors like patient’s symptoms, play a role in the decision-making process of single versus multiple-visit endodontics. However, both treatment modalities demonstrated similar success rates for the treatment of teeth with apical periodontitis^18^. Generally, the patients better tolerate and prefer single-visit root canal treatment^12^. Additionally, single-visit root canal treatment has become a common practice in non-vital cases and offers several advantages, including a decreased number of operative procedures^13^and elimination of inter-appointment leakage through temporary restorations^22^. Therefore, considering the previously mentioned advantages, root canal treatment procedures were completed in a single appointment in the present study.

Various scales and methods have been used to evaluate the severity of postoperative pain after root canal treatment including the 5-Level Pain Scale^19^, pain intensity in both numeric and verbal scores (using VAS)^20^, and the 4-point pain intensity scale^9^. In the present study, a 4-point pain intensity scale was used which is considered adequately valid and reliable^9^. A maximum 48 h time period was selected to evaluate postoperative pain, since the prevalence and severity of pain substantially decrease within the first 2 days^23^.

In the present study, the effect of three different instrumentation techniques on postoperative pain was evaluated in three different time periods. Factors that may influence the amount of extrusion of debris including apical foramen diameter, the type and quantity of irrigant solution were standardized in all groups.

To the authors’ knowledge, no data exist on the influence of instrumentation by using recently introduced ProTaper Next instruments on postoperative pain. According to the results of the present study, the modified step-back technique resulted in less postoperative pain than the other techniques. Thus, the null hypothesis was rejected. This result contrasted with previous studies^2,10,19^. Arias, et al.^2^ (2015) reported higher postoperative pain for manual instrumentation compared to rotary instrumentation. Similarly, Wei, et al.^30^ (2003) reported less postoperative pain with rotary files than with hand files. On the other hand, Pasqualini, et al.^19^ (2012) reported less postoperative pain for manual glide path preparation with K-files compared to mechanical preparation with PathFiles. These conflicting results could be related to the discrepancies of instrumentation techniques and systems used for instrumentation of root canals.

The reciprocal and rotational instrumentation techniques caused similar incidence and intensity of post-operative pain. However, the reciprocal technique demonstrated slightly higher pain scores than the rotational technique without any significance. There is a lack of studies that assess the incidence of postoperative pain when shaping with WaveOne or ProTaper Next^6,11,15^.

In the present study, the significant difference in postoperative pain between the three groups might be attributed to the extrusion of debris^8^, which is likely to depend on the instrumentation technique. Apical debris extrusion can cause periodontal ligament inflammation with higher neuropeptide concentration, and consequently peripheral sensitization characterized as hyperalgesia, allodynia, and spontaneous pain can take place^14^. In addition, a recent systematic review and meta-analysis study demonstrated that inflammatory reaction is not influenced by the number of files but the type of movement and the instrument design^6^.

A large number of studies have dealt with the effect of various root canal instrumentation techniques on the amount of the apically extruded dentinal debris and irrigant. The apical extrusion of debris following the reciprocal (WaveOne) and rotational (ProTaper Next) techniques was previously evaluated and was found to be similar for both techniques^16,26^. In contrast to these results, some previous studies reported that rotational instruments showed higher debris extrusion than reciprocal instruments^8,27^. On the other hand, Bürklein & Schäfer^5^ (2012) concluded that the full sequence rotary instrumentation was related with less debris extrusion than the reciprocal instrumentation technique. The conflicting results could be related to discrepancies in the experimental setup, design, and type of teeth used.

The results of the present study demonstrated that the mean scores of postoperative pain gradually decreased throughout the 12 h-48 h periods. None of the patients reported an increase in pain intensity from 12 h to 48 h which is in accordance with a previous finding^20^.

In conclusion, all instrumentation techniques caused postoperative pain. The mean pain scores demonstrated that all techniques produced slight pain that caused mild discomfort and no need for additional treatment. It can be concluded that the modified step-back technique can produce less pain compared to the rotational and reciprocal techniques especially in the 48 h period. The effect of instrumentation techniques on the incidence and intensity of postoperative pain should be well-evaluated.
